# An international evaluation of ultrasound vs. computed tomography in the diagnosis of appendicitis

**DOI:** 10.1186/1865-1380-4-68

**Published:** 2011-10-29

**Authors:** Betzalel Reich, Todd Zalut, Scott G Weiner

**Affiliations:** 1 Tufts University School of Medicine, Boston, MA, USA; 2 Department of Emergency Medicine, Shaare Zedek Medical Center, 12 Bayit Street, Jerusalem, 91031, Israel; 3 Tufts University School of Medicine, Tufts Medical Center, Department of Emergency Medicine, Washington Street, Boston, MA, 02111, USA; 4 Department of Emergency Medicine, Beth Israel Deaconess Medical Center, West Campus, 330 Brookline Avenue, Boston, MA 02215, USA

## Abstract

**Background:**

Abdominal computed tomography scan (CT) is the preferred radiographic study for the diagnosis of appendicitis in the United States, while radiologist-operated ultrasound (US) is often used in Israel. This comparative international study evaluates the performance of CT vs. US in the evaluation of acute appendicitis.

**Methods:**

A retrospective chart analysis was conducted at two tertiary care teaching hospitals, one in each country. Adult patients (age 18-99) with an Emergency Department (ED) working diagnosis of appendicitis between 1 January 2005 and 31 December 2006 were reviewed. Included patients had at least one imaging study, went to the OR, and had documented surgical pathology results.

**Results:**

Of 136 patients in the United States with the ED diagnosis of appendicitis, 79 met inclusion criteria for the CT cohort. Based on pathology, CT had a sensitivity of 100% (95% CI 95.4-100%). The negative appendectomy rate in patients with positive CT was 0%. Total median ED length of stay was 533 min [IQR (450-632)] and median time from CT order to completion was 184 min [IQR (147-228)]. Of 520 patients in Israel, 197 were included in the US cohort. Based on final pathology, US had a sensitivity of 68.4% (95% CI 61.2-74.8%). The negative appendectomy rate in patients with positive US was 5.5%. The median ED length of stay for these patients was 387 min [IQR (259-571.5)]. Of the patients, 23.4% had subsequent CT scans. Median time from US order to completion was 20 min [IQR (7-49)]. Both time values were *p *< 0.001 when compared with CT. We furthermore calculate that a "first pass" approach of using US first, and then performing a confirmatory CT scan in patients with negative US, would have saved an average of 88.0 minutes per patient in the United States and avoided CT in 65% of patients.

**Conclusions:**

Radiologist-operated US had inferior sensitivity and positive predictive value when compared with CT, though was significantly faster to perform, and avoided radiation and contrast in a majority of patients. A "first-pass" approach using US first and then CT if US is not diagnostic may be desirable in some institutions.

## Background

Acute appendicitis is the most common surgical emergency of the abdomen, and there are about 250, 000 new cases a year in the United States. The lifetime risk of appendicitis is approximately 8.6% in males and 6.7% in females [1]. Despite the frequency of the disease, the clinical diagnosis of appendicitis remains a diagnostic challenge [2]. Historically, classic physical findings such as pain at McBurney's point or the psoas sign have been used to make the diagnosis, though the discriminative power of classic clinical and even laboratory findings remains low [3-5]. The presence of these signs increases the likelihood of appendicitis [6], though no physical exam finding can effectively diagnose appendicitis.

An imaging study allows an objective confirmation of the diagnosis before an invasive procedure is performed. The two most common modalities in use are abdominal helical computed tomography (CT) and abdominal ultrasound (US) [7-10]. Both are considered to have acceptable sensitivities, specificities, and positive and negative predictive values, though CT has been shown to be superior in numerous studies [7-11]. The introduction of CT has led to a marked decrease in the rate of negative appendectomy, as much as 48% in one institution [12]. Compared with clinical and laboratory findings alone, the addition of CT increased diagnostic sensitivity from 91.6% to 98.3% [13].

Despite its superior sensitivity, there are at least three problems with abdominal CT. The first is that the test involves subjecting the patient to iatrogenic ionizing radiation, which carries a notable, though theoretical risk of cancer [14-16]. The second problem is that the scanners are expensive and not available in all medical practice environments, particularly in developing countries. Finally, at some facilities, administration of oral and/or rectal contrast is preferred, leading to prolonged emergency department (ED) length of stay, and when IV contrast is administered, there is a risk of allergic reaction or nephrotoxicity [17,18].

Abdominal ultrasound may offer a role in solving these problems. It is safer, relatively inexpensive, and multiple meta-analyses demonstrate a satisfactory sensitivity and positive predictive value, though inferior to that of CT [7-11]. In Israel, for example, US is often the initial imaging study of choice, followed by CT for inconclusive cases [19]. In the United States, CT is currently recommended as the first-line test in the case of suspected appendicitis, and its use is increasing [15,16,20].

In this international study, we compared the performance of CT and US in the evaluation of suspected acute appendicitis in adults in two hospitals, one in the United States and one in Israel. It is a natural experiment based on the different imaging preferences of the two countries. We evaluate the sensitivities, positive predictive values, and particularly emphasize differences in lengths of stay in the ED.

## Methods

A retrospective chart analysis was conducted in the emergency departments of two tertiary care teaching hospitals: Tufts Medical Center in the USA (annual census 39, 000) and Shaare Zedek Medical Center in Israel (annual census 60, 000). The institutional review boards at both institutions approved the study. Charts of all adult patients (age 18-99) with an ED working diagnosis of appendicitis between 1 January 2005 and 31 December 2006 were reviewed. To meet inclusion criteria, patients required the following: (1) a working diagnosis of appendicitis in the ED; (2) at least one imaging study; (3) operative management; (4) documented surgical pathology results; and (5) complete chart information for appropriate data abstraction. Patients meeting inclusion criteria were divided into two cohorts based on whether they were evaluated in the United States (CT cohort) or in Israel (US cohort).

Surgical pathology was considered the gold standard for calculation of sensitivities of CT and US. Radiographic results were evaluated in order to calculate sensitivity and positive predictive values (PPV). Chart information was abstracted to determine negative appendectomy (NA) rates, clinical and laboratory findings, and the following chronological components: time from ED admission to imaging ordered; imaging ordered to imaging completed; imaging completion to ED discharge; and total ED length of stay.

The charts of all patients were contained on a computerized charting program, Tufts Medical Center program EDIS, Medhost (Addison, TX), and the Shaare Zedek Medical Center home-built program (Jerusalem, Israel). Imaging studies and pathology reports at Tufts Medical Center were available on Soarian (Siemens Medical Solutions, Malvern, PA). Of note, the data abstractor had formal medical training and was fluent in both Hebrew and English.

### Ultrasound examination

Color Doppler sonography of the right lower quadrant was performed using the graded compression technique with a Phillips HDT 5000 linear 1-5 MHz transducer, according to body size. Visualization of an incompressible blind-ended appendix measuring more than 6 mm in diameter with additional positive findings, including echogenic periappendicular fat, hyperemic appendiceal walls, appendicolith, pericecal fluid, or abscess, was diagnostic of appendicitis. The US report was read as positive, negative, or not visualized (NV) for acute appendicitis.

### Contrast-enhanced MDCT examination

CT exams were performed using a multi-slice CT scanner (SOMATON Sensation or Definition, Siemens Medical Solutions USA, Inc., Malvern, PA). The most common technique involved the use of triple contrast (oral, rectal, and IV). Patients were initially prepped with 1 l of oral and 300 cc of rectal contrast (Bracco Diagnostics Inc., Princeton, NJ), followed by 145 cc of Isovue-300 IV contrast at a rate of 2 cc/s just prior to the scan. Serial 3-mm axial images were obtained from the diaphragm through the perineum. Additional delayed images were obtained through the lower abdomen after the patient was asked to lay on the right side for 10 min. Visualization of an appendix measuring more than 6 mm in diameter with additional positive findings, including periappendicular fat stranding, cecal wall thickening, appendicolith, abscess, or phlegmon, was diagnostic for appendicitis. The CT report was read by the radiologist as positive or negative for appendicitis.

### Radiology

In the United States, CT scans were performed in the Department of Radiology by qualified technicians and read by senior level radiology residents. In Israel, ultrasounds were performed and read by trained radiologists in a room adjacent to the ED. Subsequent CT scans were performed in the Department of Radiology for a portion of the patients. All studies were officially read by senior resident or attending radiologists at both sites at the time of imaging.

### Statistical analysis

Sensitivity, PPV, and NA were calculated. When calculating sensitivity, ultrasounds in which the appendix was not visualized were considered to be negative. Statistical analysis was performed using JMP 8 (SAS, Cary, NC).

## Results

**Figure 1:**
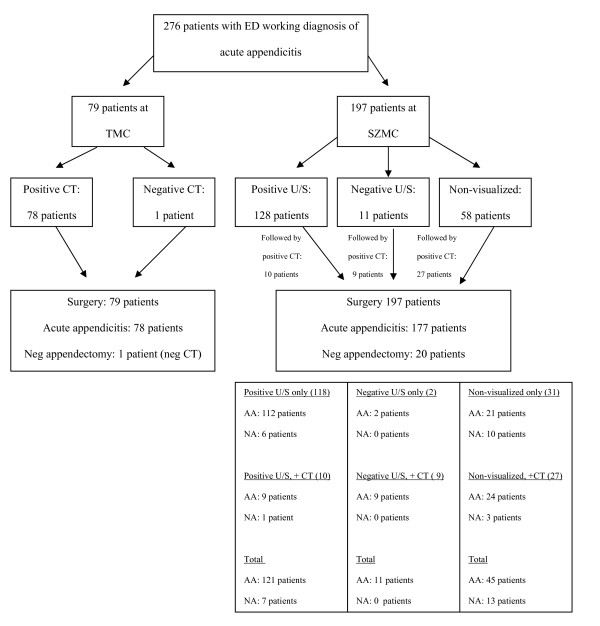
**Imaging and surgical results of suspected acute appendicitis**.

Of patients in the United States, 79 of 136 (58%) met inclusion criteria and were included for analysis. Of the Israeli patients, 197 of 520 (38%) were similarly included. Patient characteristics of the two cohorts are shown in Table 1, and patient outcomes are shown in Table 2. A flow chart including imaging and surgical results is displayed in Figure 1.

**Table 1:** Characteristics of CT and US cohorts

Characteristic	CT cohort (*N *= 79)	US cohort (*N *= 197)	*P*
Age (years)			
Mean (95% CI), *n*	40.2 (36.8-43.6), 75	30.2 (28.6-31.9), 197	< 0.001
Gender			
Female % (*n*)	38.7% (29)	59.9% (117)	0.003
Male % (*n*)	61.3% (46)	40.1% (80)	
Temperature (°C)			
Mean (95% CI), *n*	36.8 (36.6-36.9), 78	37.1 (37.0-37.2), 186	< 0.001
Heart rate (beats/min)			
Mean (95% CI), *n*	82.7 (79.2-86.2), 79	87.4 (85.0-89.9), 178	0.028
Systolic BP (mmHg)			
Mean (95% CI), *n*	126.6 (122.6-130.6), 79	118.4 (116.2-120.7), 180	< 0.001
Diastolic BP (mmHg)			
Mean (95% CI), *n*	71.6 (69.1-74.1), 79	69.9 (68.3-71.6), 180	0.268
White blood count (10^3^/mm^3^)			
Mean ± 95% CI, *n*	12.6 (11.6-13.5), 78	13.5 (12.9-14.1), 197	0.095

*Means were compared with two-tailed t-test. Gender was compared with chi-square test.

**Table 2:** Outcomes of CT and US cohorts

Outcome	CT cohort (*N *= 79)	US cohort (*N *= 197)	
Sensitivity			
%, ± 95% CI	100% (94.2-100%)	68.4% (60.9-75.0%)	
PPV			
%, ± 95% CI	100% (94.2-100%)	94.5% (88.6-97.6%)	
Neg appendectomy (after positive imaging)	0% (0 of 78)	5.5% (7 of 128)	*p *= 0.049
Times (min)			
Admission to imaging ordered			
Mean (95% CI)	142.6 (122.6-162.6)	158.4 (139.3-177.5)	*p *= 0.870
Median (IQR)	130.0 (74.0-198.0)	114.0 (74.5-193.5)	
Imaging ordered to complete			
Mean (95% CI)	194.2 (177.9-210.5)	38.2 (31.1-45.3)	*p *< 0.001
Median (IQR)	184.0 (147.0-228.0)	20.0 (7.0-49.0)	
Imaging complete to ED disposition			
Mean (95% CI)	222.7 (187.5-257.8)	251.7 (221.2-282.3)	*p *= 0.891

Median (IQR)	192 (141.0-266.0)	193.0 (101.0-312.5)	

Admission to ED disposition			

Mean (95% CI)	559.5 (518.6-600.4)	448.3 (410.9-485.7)	*p *< 0.001

Median (IQR)	533.0 (450.0-632.0)	387.0 (259.0-571.5)	

*Means were compared with two-tailed t-test. Negative appendectomy rate was compared with two-tailed Fisher's exact test.

Of the 79 patients in the United States that underwent CT scans, 78 had a positive scan and one had a negative scan. All 79 patients underwent surgery. All 78 positive scans were confirmed to be appendicitis by surgical pathology. The one negative scan underwent surgery based on strong clinical suspicion, but this patient had a negative appendectomy.

Of the 197 patients in Israel that initially underwent a US, 139 had a diagnostic scan, of which 128 were positive and 11 were negative, while 58 patients had a non-diagnostic scan in which the appendix was not visualized. Forty-six (23.4%) adjunct CT scans were performed after the initial US. These included 10 CT scans after a positive US, 9 scans after a negative US, and 27 scans after a non-diagnostic US. All 197 patients underwent surgery. One hundred seventy-seven patients were found to have appendicitis based on pathology. Of these confirmed cases of appendicitis, 121 patients had a positive US, 11 had a negative US, and 45 patients had a non-diagnostic US. Twenty patients had a negative appendectomy, including 7 patients that had a positive US and 13 that had a non-diagnostic US.

The sensitivity of CT in the United States was 100% (78 of 78), and the positive predictive value was 100% (78 of 78). The negative appendectomy rate was 1.2% (1 of 79). However, that patient had a negative CT prior to surgery, and for the purpose of this study, only negative appendectomies following positive CTs were included in the calculation of the NA. Therefore, the NA was actually 0.0%.

The sensitivity of US in Israel was 68.4% (121 of 177), and the positive predictive value was 94.5% (121 of 128). The negative appendectomy rate was 10.2% (20 of 197). However, following a positive US, the NA rate was 5.5% (7 of 128). These statistical data are demonstrated in Table 2.

In the United States, mean time from admission to the imaging order was 142.6 min (95% CI 122.6-162.6). Time from the imaging order to completion was 194.2 min (95% CI 177.9-210.5). Time from imaging completion to disposition was 222.6 min (95% CI 187.5-257.8). Total time from admission to disposition was 559.4 min (95% CI 518.6-600.4).

In Israel, mean time from admission to the imaging order was 158.4 min (95% CI 139.3-177.5). Time from the imaging order to completion was 38.2 min (95% CI 31.1-45.3). Time from imaging completion to disposition was 251.7 min (95% CI 221.2-282.3). Total time from admission to disposition was 448.3 min (95% CI 410.9-485.7). All times for both sites are shown in Table 2.

## Discussion

Abdominal imaging is currently indicated in all but the most straightforward cases of appendicitis [17]. However, the choice of which study to use--either US or CT--remains a point of contention. In children, ultrasound is a viable and commonly used choice, though in adults, the choice is less clear [21]. CT clearly has its advantages, with sensitivity approaching 100% and the ability to perform the study in a way that is not operator dependent, and in patients in which ultrasound is difficult to perform, such as those who are obese [19,21]. However, the risks of contrast administration, exposure to ionizing radiation, and cost are all limiting factors [14-18]. With an estimated 2% of future cancers being caused just by CT scans, clinicians need to determine ways to reduce this exposure [22].

Despite the increase in CT usage in the United States, ultrasound continued to serve as the primary modality in many hospitals in Israel. The reasoning behind the use of ultrasound in Israel is likely multi-factorial. In 2000, only 38 CT scanners were operating in Israel, representing one of the lowest ratios of CT per population in the developed world [23]. In addition, healthcare in Israel is socialized and cost-effectiveness is stressed. A CT scan of the abdomen and pelvis costs almost three times more than an ultrasound [19]. With constant pressure to cut costs where possible without causing harm to the patient, US is often used as the primary modality for the workup of appendicitis. These differences in practice patterns between the two countries pushed us to explore the possibility of integrating ultrasound into the workup of suspected acute appendicitis in adult patients in the United States. While the sensitivity is inferior, US is known to be useful in children and pregnant patients, and is the primary modality for these subset of patients based on the American College of Radiology guidelines [24].

Multiple studies have directly compared CT and US accuracy in the diagnosis of appendicitis. A meta-analysis of prospective studies of the accuracy of CT and US in the diagnosis of acute appendicitis in adults and adolescent patients, including four studies directly comparing the two, showed that CT was superior to US. CT sensitivity was 0.94 (95% CI: 0.91 to 0.95) and specificity 0.95 (95% CI: 0.93 to 0.96), while US sensitivity was 0.86 (95% CI: 0.83 to 0.88) and specificity 0.81 (95% CI: 0.78 to 0.84) [7]. Other studies have shown that modern CT scanners have a sensitivity of 90-100%, a specificity of 91-99%, and a positive predictive value of 95-97%. In contrast, a carefully performed US has a sensitivity of 75-90%, a specificity of 86-100%, and a positive predictive value of 89-93% [20].

Despite the established superiority that CT has over ultrasound for the diagnosis of appendicitis, recent studies have advocated for a first-line ultrasound approach with adult patients presenting with possible appendicitis [9,19,25,26]. Lameris et al. [3] recommend a conditional CT strategy, with initial US in adult patients presenting with acute abdominal pain, including suspected appendicitis, and CT only after negative or inconclusive US. With this strategy only 50% of patients required CT scans with a low NA rate. Gaitini et al. [19] found that routine referral of adult patients with clinical suspicion of acute appendicitis to color Doppler US and selected referral to CT based on US results and clinical judgment improved diagnostic accuracy and therapeutic management. Poortman et al. [25] also concluded that a diagnostic pathway including an initial US and complimentary CT in patients with negative or inconclusive US results yields a high diagnostic accuracy in the management of acute appendicitis without adverse events. The message of these studies is the same: the positive predictive value of US is excellent; if the appendix is visualized and abnormal, the patient should go to surgery. If the appendix is not visualized, then the patient should have a CT. This approach has clearly been shown to be cost effective and safe in children, and we posit that it may be in adults as well [26]. This stepwise approach in the pediatric population was also supported by Ramarajan et al., who found that by employing US first in the diagnostic pathway of appendicitis, radiation exposure may be substantially reduced without a decrease in safety or efficacy [27].

Our study is unique in that it studies a system where ultrasound is a common modality for the workup of appendicitis and compares it to the well-established CT protocol used in the United States. In this study we found that CT is superior to US for the diagnosis of acute appendicitis in adults. CT sensitivity was higher than US (100% vs. 68.4%) as was PPV (100% vs. 94.5%). Negative appendectomies after positive scanning were non-existent with CT and 5.5% with US. These findings are not surprising and are similar to those in the previous literature.

In this study we discovered that the mean time it takes to perform an ultrasound in Israel is significantly faster than a CT scan in the United States (38.2 vs. 194.2 min). One hundred twenty-eight of 197 patients (65.0%) had positive ultrasounds in Israel. Employing a purely hypothetical calculation, ignoring the system-wide differences that exist, if we were to apply a theoretical "first-pass" US approach in our United States hospital and first obtain an ultrasound in all of these patients, we would come up with the following calculation: the 79 patients in the cohort would have had an ultrasound at 38.2 min each, for a total of 3, 017.8 min. Assuming that 65% would be positive (as in the Israel cohort), then the remaining 35% of patients (27.65

patients) would then have a CT scan. This total time would be an additional 5, 369.6 min for these patients. Therefore, the total time taken by this theoretical pathway would be 3, 017.8 + 5, 369.6 min = 8, 388.4 min, or 106.2 min/patient. We already know that the mean time for obtaining a CT in this cohort was actually 194.2 min. By performing the hypothetical first-pass ultrasound, we would have saved 194.2 -106.2 = 88.0 min per patient.

Multiple authors have attempted to reduce CT scan time. Berg et al. [18] found that administering rectal contrast only without waiting for a full oral contrast preparation safely shortened patient throughput time. A recent meta-analysis also showed that the diagnostic accuracy of a non-contrast CT scan is sufficient, with a pooled sensitivity and specificity of 92.7% (95% CI 89.5-95.0%) and 96.1% (95% CI 94.2%-97.5%), respectively [28]. However, US maintains the advantage of being quick, inexpensive, and potentially portable. Other studies have shown reliable exams when performed by surgeons, emergency physicians, or even emergency medicine residents at the bedside [29-32]. These studies all advocate further testing if the US is negative or indeterminate.

### Limitations

This study has limitations that must be considered when interpreting its results. The first is that this was a retrospective study. We were reliant on the presence of correct data in the medical chart. A large number of patients in Israel had to be excluded for lack of complete clinical information. The second obvious major limitation is that we compared two different imaging modalities in two different countries with clearly different medical systems, patient populations, and cultures. This step was necessary to make such a comparison between two systems, but must be considered. Clearly, a prospective, randomized trial of CT and US performance within the same country would be ideal. Finally, the patient populations that were compared were different, with those in Israel tending to be younger and more likely to be female than those in the United States cohort.

## Conclusions

Radiologist-operated US had inferior sensitivity and positive predictive value when compared with CT, though was significantly faster to perform, and avoided ionizing radiation and contrast in a majority of patients. As a means of balancing test performance with side effects and ED patient throughput times, a "first-pass" approach using US first and then CT if US is not diagnostic may be desirable.

## Competing interests

The authors declare that they have no competing interests.

## Authors' contributions

BR carried out chart review and drafted the manuscript. TZ conceived of the study from the Israeli standpoint, participated in the design of the study, and helped draft the manuscript. SW conceived of the study from the United States standpoint, participated in the design of the study, performed the statistical analysis, and participated in the drafting of the manuscript. All authors read and approved the final manuscript.
